# Effects of *Dendrobium officinale* Leaf Powder on Bone Health and Bone Metabolism in Laying Hens

**DOI:** 10.3390/ani16020329

**Published:** 2026-01-21

**Authors:** Yutao Wu, Bingji Xu, Haoxin Zhang, Wen Ge, Ayong Zhao, Han Wang, Feifei Yan

**Affiliations:** 1 Key Laboratory of Applied Technology on Green-Eco-Healthy Animal Husbandry of Zhejiang Province, College of Animal Science and Technology & College of Veterinary Medicine of Zhejiang A&F University, Hangzhou 311300, China; 2 Hangzhou Lin’an AiGe Poultry Co., Ltd., Hangzhou 311300, China

**Keywords:** *Dendrobium officinale*, bone strength, bone density, bone metabolism

## Abstract

*Dendrobium officinale* Kimura et Migo (*D. officinale*) is a traditional Chinese medicinal herb with recognized anti-inflammatory, antioxidant, and immunomodulatory properties. This study evaluated whether dietary supplementation with *D. officinale* leaf powder could influence bone mass, mechanical strength, and molecular markers of bone metabolism in caged laying hens. A total of 192 healthy 19-week-old Jinghong No. 1 hens were randomly assigned to three dietary groups: a control group fed a basal diet and two treatment groups supplemented with 1200 or 3600 mg/kg of *D. officinale* leaf powder for 16 weeks. Tibial and femoral bone strength and mineral density did not differ significantly among treatments (*p* > 0.05). However, tibial breaking strength displayed upward trends in both supplemented groups (*p* = 0.08), and similar tendencies were observed for femoral bone mineral content and bone density (*p* = 0.08). At the molecular level, dietary supplementation produced selective changes in gene expression. The low-dose diet significantly increased VEGFA expression (*p* < 0.05), whereas the high-dose diet resulted in significantly higher TGF-β1 expression (*p* < 0.05). Several other genes related to bone formation, bone resorption, or cytokine signaling exhibited numerical increases but did not reach statistical significance. These findings indicate that *D. officinale* leaf powder may modulate bone metabolic processes at the transcriptional level, although these molecular alterations were not accompanied by significant improvements in bone mass.

## Simple Summary

Laying hens kept in cages often experience bone weakness, which increases the risk of fractures and can negatively affect their productivity and welfare. In this study, we evaluated whether adding leaves of *Dendrobium officinale*, a traditional medicinal plant, to the diet could help support bone health. Although 16 weeks of supplementation did not lead to clear improvements in bone mass, including bone strength and bone density, several indicators showed upward numerical trends. In addition, the dietary supplement influenced key molecular markers of bone metabolism. Notably, hens receiving the low-dose diet exhibited higher expression of VEGFA, a gene involved in vascular development within bone tissue, while the high-dose diet significantly increased the expression of TGF-β1, a cytokine linked to bone remodeling. Other genes showed small numerical increases but no statistical differences. These results suggest that *D. officinale* may help promote healthy bone metabolic processes at the molecular level. As a natural plant-derived additive, it may offer a safe and sustainable approach to improving the health and welfare of laying hens.

## 1. Introduction

In laying hens, the skeleton not only provides structural support but also serves as a crucial reservoir for calcium. Approximately 30–35% of the calcium needed for eggshell formation is mobilized from bone tissue [1], placing considerable metabolic stress on the skeletal system during peak production. Under prolonged cage housing, hens are prone to osteoporosis and cage layer fatigue, which can lead to fractures, paralysis, and reduced egg production and quality [2,3]. Therefore, nutritional strategies that promote skeletal health are essential to ensure productivity and animal welfare in modern poultry systems.

*Dendrobium officinale* Kimura et Migo (*D. officinale*) is a traditional Chinese medicinal herb renowned for its antioxidant, anti-inflammatory, immunomodulatory, and anti-osteoporotic properties [4,5,6]. Importantly, the bone-protective evidence reported to date has involved different preparations and constituents of *D. officinale*. For example, a crude water extract of *D. officinale* (a mixture containing multiple components) was reported to attenuate ovariectomy-induced bone loss and inhibit RANKL-induced osteoclast differentiation [7]. More recent mechanistic studies have focused on defined constituents, including *D. officinale* polysaccharides [8] and the alkaloid dendrobine [9], which were shown to promote osteogenic activity and/or mitigate glucocorticoid-induced bone damage. Together, these findings suggest that *D. officinale* may influence bone remodeling through pathways related to osteogenesis and osteoclastogenesis, including signaling linked to the OPG/RANKL/RANK axis and osteogenic regulators.

Although most studies have focused on the stems of *D. officinale*, its leaves are a low-cost by-product and represent the non-medicinal part of the plant that is more practical, food-grade resource for feed application. Available studies indicate that *D. officinale* leaves contain comparable levels of bioactive compounds such as polysaccharides, flavonoids, and alkaloids [10]. These compounds also exhibit immunomodulatory and antioxidative effects [11], making the leaves a promising feed additive for poultry. From a production perspective, using whole-leaf powder rather than purified extracts is more feasible for routine dietary supplementation. Our previous research demonstrated that dietary inclusion of *D. officinale* leaves improved egg quality parameters and reduced the incidence of damaged eggs in laying hens [12]. Although the bioactive composition of plant materials can vary across different batches due to factors such as plant origin and processing conditions, the types of bioactive compounds in the leaves remain relatively consistent. Therefore, in the present study, we focused on evaluating the in vivo efficacy of whole-leaf powder, interpreting the potential mechanisms based on existing literature. 

In poultry, bone metabolism is orchestrated by complex molecular networks involving growth factors (e.g., VEGFA), inhibitors of bone resorption (e.g., OPG), matrix-degrading enzymes (e.g., MMP9), and transcriptional regulators (e.g., RUNX2) [13,14]. Modulating these pathways through functional plant-based additives may represent an effective non-pharmacological approach to improve skeletal health.

Therefore, we hypothesized that dietary supplementation with *D. officinale* leaf powder would improve skeletal health in caged laying hens by enhancing osteogenic activity and/or attenuating bone resorption, potentially through modulation of bone remodeling–related markers and pathways (e.g., the OPG/RANKL axis and osteogenic regulators). Accordingly, the current study aimed to investigate the effects of dietary supplementation with *D. officinale* leaf powder on bone strength, bone mineral density, and the expression of bone metabolism-related genes in caged laying hens. By targeting key markers of osteogenesis and bone resorption, this research seeks to evaluate the potential of *D. officinale* leaves as a novel nutritional strategy to support skeletal homeostasis in poultry production.

## 2. Materials and Methods

### 2.1. Animals, Diets, and Sample Collection

This experiment was conducted at AiGe Poultry Industry Co., Ltd. (Hangzhou, Zhejiang Province, China). Fresh leaves of *D. officinale* were sourced from Hangzhou Zhenheng Biotechnology Co., Ltd. (Hangzhou, Zhejiang Province, China). The leaves were oven-dried at 60 °C and ground into fine powder (60-mesh sieve) for dietary inclusion.

A total of 192 healthy 19-week-old Jinghong No. 1 laying hens with comparable body condition and baseline production status were randomly assigned to three dietary treatments. Each treatment consisted of 8 replicates with 8 birds per replicate. The control group (CON) received a basal diet, while the two experimental groups were fed the basal diet supplemented with either 1200 mg/kg (DO-L) or 3600 mg/kg (DO-H) of *D. officinale* leaf powder. The feeding trial lasted for 16 weeks. The composition and nutritional content of the basal diet are presented in Table 1. Proximate nutrient values of the basal diet were calculated from the formulation and standard ingredient composition tables. Accordingly, the listed ash content reflects the calculated contribution of corn, soybean meal, and limestone, and the actual ash level may be slightly higher due to the mineral fraction of the premix. Birds had ad libitum access to feed and water throughout the trial.

At the end of the experiment, one hen per replicate was randomly selected for sample collection (n = 8). Following humane euthanasia via cervical dislocation, both legs were excised. Bone tissues were collected from the proximal epiphysis of the left tibia, immediately snap-frozen in liquid nitrogen, and stored at −80 °C for subsequent gene expression analysis. The surrounding muscle and connective tissues of the right femur and tibia were carefully removed, and the cleaned bones were stored at −20 °C for bone strength and density measurements.

### 2.2. Measurement of Bone Traits

The morphological dimensions of the tibia and femur were recorded using a high-precision digital caliper (Guilin Guanglu Measuring Instrument Co., Ltd., Guilin, China). For each bone, both the vertical and horizontal external diameters at the midshaft were measured and averaged to determine mean bone width, while bone length was measured from the proximal to distal ends.

Bone mineral parameters, including bone mineral content (BMC), bone area, and BMD, were assessed by dual-energy X-ray absorptiometry (DEXA; Medikors Inc., Seongnam-si, Republic of Korea). The mechanical integrity of the tibia and femur was evaluated through a three-point bending test using a universal testing machine (LR10K PLUS, Lloyd Instruments Ltd., Hampshire, UK). Each bone was placed on two supporting points, and a downward force was applied at the midpoint at a constant loading rate of 10 mm/min until fracture occurred. The resulting load–deformation data were processed with NEXYGEN Plus software (version 3.0) to obtain stiffness (N/m) and breaking strength (N).

### 2.3. Bone Metabolism and Cytokine mRNA Expression

Total RNA was isolated from bone tissue using the RNAiso Plus (Takara Bio Inc., Dalian, China) according to standard procedures. Reverse transcription was carried out using a commercial cDNA synthesis kit (ABM, Richmond, BC, Canada), following the manufacturer’s protocol. Target gene sequences related to bone metabolism and inflammatory cytokines in Gallus gallus were retrieved from the NCBI database (https://www.ncbi.nlm.nih.gov/, accessed on 30 June 2023). Specific primers were designed using Primer Premier 5.0 software and are listed in Table 2. Quantitative real-time PCR was conducted with the TB Green Premix Ex Taq II kit (RR420A, Takara Bio Inc., Dalian, China) to assess gene expression levels. Relative mRNA expression was calculated using the 2^−ΔΔCt^ method, with *GAPDH* used as the internal reference gene.

### 2.4. Statistical Analysis

All data were analyzed using SAS (version 9.4, SAS Institute Inc., Cary, NC, USA). Prior to analysis, the data were checked for normality and homogeneity of variances. A mixed model was used for each variable using the PROC MIXED procedure. Least squares means (LSMeans) for each treatment were obtained using the LSMEANS statement, and differences among means were evaluated with Tukey–Kramer adjustment for multiple comparisons. Results are presented as LSMeans ± SEM. Differences were considered statistically significant at an adjusted *p* < 0.05, and 0.05 ≤ *p* < 0.10 was interpreted as a tendency.

## 3. Results

### 3.1. Bone Morphometry

The dietary supplementation of *D. officinale* leaf powder had no statistically significant impact on tibial or femoral length and width across treatment groups (*p* > 0.05; Table 3). Both low (DO-L) and high (DO-H) inclusion levels of *D. officinale* did not alter bone size compared to the control.

### 3.2. Bone Mineral Density

Although not statistically significant, hens in the DO-L and DO-H groups showed a trend toward increased BMC and BMD in the tibia compared to the control group (*p* = 0.08; Table 4). No significant differences were observed in bone area among the three groups (*p* > 0.05).

### 3.3. Bone Mechanical Properties

Tibial and femoral stiffness was numerically higher in both the DO-L and DO-H groups compared to the control, but the differences were not statistically significant (*p* > 0.05; Table 5). Tibial breaking strength also showed numerical increases of approximately 76% and 61% in the DO-L and DO-H groups, respectively, yet these changes did not reach significance (*p* > 0.05; Table 5). In the femur, breaking strength exhibited upward trend (*p* = 0.08; Table 5), particularly in the DO-L group, which showed increase of 138%, compared to the control.

### 3.4. Gene Expression Related to Bone Metabolism

Expression of genes related to bone metabolism exhibited limited responses to the dietary treatments (Table 6). Among all targets measured, VEGFA expression was significantly affected by treatment (*p* = 0.03), with the DO-L group showing a marked increase relative to the control, whereas DO-H exhibited a moderate but non-significant elevation. For the remaining bone formation and bone resorption associated genes, including OPG, RUNX2, BMP2, MMP9, CTSK, and RANKL, no statistically significant differences were detected among treatments (*p* > 0.05).

### 3.5. Cytokine Gene Expression

The dietary addition of *D. officinale* had a relatively limited impact on cytokine gene transcription in the tibial epiphysis (Table 7). Among the cytokines assessed, TGF-β1 was the only gene that responded significantly to treatment, with the DO-H group showing higher mRNA levels than the control and DO-L hens (*p* = 0.02). A similar trend was showed in the expression of TGF-β3, but this change did not achieve statistical significance (*p* = 0.10). For the other cytokines examined, including IL-1β, IL-2α, IL-4, IL-6, IL-10, and TNF-α, dietary treatment did not lead to measurable differences (*p* > 0.05).

## 4. Discussion

### 4.1. Effects of D. officinale Supplementation on Bone Health

Bone serves not only as a structural framework but also as a reservoir for critical minerals such as calcium and phosphorus [15], which are essential for eggshell formation during the laying cycle. In modern egg production systems, prolonged laying periods and intensive housing conditions have increased the incidence of skeletal disorders, such as cage layer fatigue and osteoporosis, making bone health a critical welfare and productivity concern [16]. Adequate mineralization and structural integrity of bones are closely linked to reduced egg breakage and improved eggshell quality, particularly in extended production cycles [17].

In the present study, dietary supplementation with dried *D. officinale* leaf powder did not produce significant differences in BMD or bone strength in laying hens. However, numerical increases in BMD and mechanical indices (e.g., stiffness and breaking strength) were observed in the treatment groups, suggesting a potential but non-significant benefit. These findings are consistent with previous studies that report exploratory tendencies in the effects of *D. officinale* on bone parameters, though statistical significance was not reached (*p* ≈ 0.08–0.14). Therefore, the results should be viewed as exploratory evidence rather than definitive conclusions.

These findings contrast with those reported in mammalian models, where ethanol or aqueous extracts of *D. officinale* stems enhanced bone microarchitecture and mineralization in glucocorticoid- or estrogen-deficiency models [7,8,9]. For example, a 1.05 g/kg dose of stem extract was shown to improve femoral trabecular parameters, suggesting a potential anabolic effect in ovariectomized mice. This discrepancy between our study and mammalian reports may result from differences in extract composition, purification level, and effective dosage, as mammalian studies generally use concentrated stem extracts or isolated polysaccharides rather than whole-leaf powder. Based on an extraction rate of approximately 23.5% [7], the effective intake in the present study was approximately 0.07 g/kg. This is notably lower than the dose used in mammalian studies, and the lower bioactive compound content in leaf tissue compared to stems may further limit efficacy. Moreover, differences in physiological state, age, and metabolic demand between normal laying hens and osteoporotic rodent models likely contribute to these divergent outcomes. These findings align with observations by Zhang et al. [18], who reported that freeze-dried *D. officinale* preparations had stronger immunomodulatory effects than crude powder, emphasizing the importance of preparation methods in determining efficacy.

### 4.2. Expression of Bone Metabolism-Related Genes in Response to Dietary D. officinale

Bone homeostasis is maintained through a dynamic balance between osteoblastic bone formation and osteoclastic resorption, mediated by tightly regulated signaling networks [19]. In the present study, dietary *D. officinale* produced selective transcriptional effects on markers related to bone metabolism. Among the osteogenic markers examined, only VEGFA exhibited a significant treatment response, with hens receiving the low-dose diet showing markedly higher expression. VEGFA is recognized as a key mediator coupling angiogenesis with endochondral ossification, and its upregulation suggests enhanced vascular support for bone remodeling [20]. Although RUNX2, OPG, and BMP2 did not differ statistically among treatments, their numerical increases in the supplemented groups may suggest a mild stimulatory trend on osteoblast-associated pathways. These findings should be interpreted as exploratory tendencies, given the statistical non-significance (*p* > 0.05).

For genes linked to bone resorption, including RANKL, CTSK, and MMP9, dietary *D. officinale* supplementation did not produce significant changes, although the high-dose diet generally led to higher mean expression levels. These molecules are central regulators of osteoclast differentiation and matrix degradation [19,21], and the non-significant yet upward directional changes may reflect a modest activation of resorptive processes, but caution is needed in interpreting these trends as definitive biological effects. Taken together, the pattern of increased pro-osteogenic and pro-resorptive gene expression—albeit largely not significant—suggests possible bone turnover changes. However, these changes were not large enough to produce measurable improvements in bone density or mechanical strength, and caution is needed in interpreting these findings as definitive biological effects.

These results differ from several earlier studies in which components derived from *D. officinale* showed clear regulatory effects on osteoclastogenesis and osteogenesis in vitro. Han et al. reported that *D. officinale* polysaccharides inhibited RANKL-induced osteoclast differentiation and downregulated osteoclastic gene expression in a dose-dependent manner [22]. Similar inhibitory effects on osteoclast formation were observed in the in vitro studies conducted by Wang et al. [7,8], while Li et al. [9] demonstrated that dendrobine enhanced osteogenic differentiation of bone marrow stromal cells under glucocorticoid challenge. However, differences in experimental design, including the use of stem extracts versus whole-leaf powder, may contribute to the divergent results observed in our study.

However, the cellular responses reported in these studies were consistent with their corresponding mammalian in vivo findings, particularly in ovariectomized or glucocorticoid-induced osteoporotic models. The differences observed in our study are likely due to variations in experimental context, such as the use of concentrated stem extracts in mammalian studies versus whole-leaf powder in the present trial. Additionally, differences in physiological state and metabolic demand between osteoporotic rodent models and healthy, early-laying hens may contribute to the observed discrepancies.

In addition, in vivo research on *D. officinale* and skeletal metabolism in poultry remains scarce, limiting direct comparisons across studies. The functional relevance of slightly elevated bone turnover in healthy, non-osteopenic hens is not yet fully understood and may depend on physiological stage or skeletal condition. Further investigations using higher supplementation levels, purified bioactive components, or models with compromised bone status (such as hens in the late-laying phase) are warranted to clarify the potential of *D. officinale* as a functional feed additive for improving skeletal health. Additionally, future studies should include both single-component and combined supplementation studies to better understand the synergistic effects of these bioactive compounds when used together.

### 4.3. Cytokine Regulation and Its Link to Bone Metabolism

Cytokines are key mediators in immune regulation and have increasingly been recognized for their role in bone remodeling [23]. In the present study, dietary supplementation with *D. officinale* leaf powder produced selective effects on cytokine gene expression in bone tissue. Among the cytokines examined, only TGF-β1 showed a significant response, with higher expression observed in hens receiving the high-dose diet. TGF-β family members are well established as regulators of osteoblast activity and extracellular matrix production, and the elevated TGF-β1 expression suggests a potential stimulatory effect on osteogenic signaling [24].

Other cytokines, including IL-4, IL-6, IL-10, TNF-α, and TGF-β3, showed numerical but non-significant increases. IL-4 and IL-10 are generally associated with inhibition of osteoclastogenesis and support for bone formation [23], whereas IL-6 has context-dependent roles and can influence both osteoblasts and osteoclasts [25]. TNF-α is a cytokine with strong pro-inflammatory and osteoclast-activating properties. Although modest, these upward trends suggest that *D. officinale* may exert mild immunomodulatory effects relevant to bone remodeling. However, these trends were not statistically significant, and further studies with larger sample sizes are needed to confirm these findings. Similar anti-inflammatory patterns have been reported in mammalian studies, where *D. officinale* polysaccharides reduced pro-inflammatory cytokines such as TNF-α, IL-6, and IL-1β and increased IL-10 across cyclophosphamide-treated mice and epileptic rat models [11,26], supporting a role for immunoregulation in its bioactive property.

Nevertheless, a limitation of this study is that the bioactive constituents of the *D. officinale* leaf powder were not chemically quantified; therefore, mechanistic interpretation is based on observed in vivo responses and evidence from prior literature. In addition, because whole-leaf powder represents a complex mixture of bioactive compounds, quantification of individual constituents alone would not be sufficient to elucidate their interactive effects. Furthermore, it is important to emphasize that the primary objective of this research was to evaluate the practical application of *D. officinale* leaf powder as a functional feed additive in poultry production, rather than as a highly standardized pharmacological agent. As a natural, whole-plant material, its bioactive composition inherently possesses a degree of natural variability. This ‘whole-component’ approach reflects the real-world conditions under which such additives are utilized in the poultry industry, where the focus is on the collective biological efficacy of the complex mixture in supporting animal health and welfare. Future studies should combine phytochemical characterization with experimental designs comparing single-component and combined supplementation to better clarify the mechanisms involved.

## 5. Conclusions

Dietary supplementation with *D. officinale* leaf powder did not lead to significant changes in bone density or mechanical strength in laying hens. However, it did modify several molecular markers involved in bone formation, resorption, and remodeling. In particular, the low-dose diet elevated VEGFA, while the high-dose diet increased TGF-β1, suggesting a potential stimulatory effect on bone metabolic signaling. These transcriptional responses indicate that *D. officinale* may influence bone turnover processes; however, the lack of significant changes in bone density and mechanical strength highlights the need for caution in interpreting these findings. Future studies employing higher inclusion levels, purified bioactive components, or experimental models with greater skeletal demands—such as hens in the late-laying phase—are needed to further clarify its potential as a functional feed additive for supporting skeletal health in poultry.

## Figures and Tables

**Table 1:** Basic diet composition and nutrient composition.

Parameter	Content
Dietary composition, %	
Corn	62.48
Soybean meal	25.79
Limestone	7.93
Premix ^1^	3.00
Soybean oil	0.80
Nutrient composition	
Metabolic energy, MJ/kg	11.63
Total phosphorus, %	0.40
Calcium, %	3.2
Crude protein, %	16.07
Crude fiber, %	2.92
Ash, %	10.15
Organic matter, %	89.85
Crude fat, %	3.40
Lysine, %	0.89
Methionine, %	0.32
Threonine, %	0.61
Tryptophan, %	0.16

^1^ The premix is provided for each kilogram of feed: 14,400 IU of vitamin A, 5600 IU of vitamin D3, 32 mg of vitamin E, 32 mg of vitamin K, 2.4 mg of vitamin B1, 12 mg of vitamin B2, 4 mg of vitamin B6, 54 mg of niacinamide, 4 mg of pantothenic acid, 0.4 mg of iodine, 50 mg of iron, 96 mg of zinc, 100 mg of manganese, 6 mg of copper, and 0.48 mg of selenium.

**Table 2:** Primer sequences (5′ → 3′).

Gene	Gene Accession Number	Primer Sequence	Amplicon Size (bp)
*GAPDH*	NM_204305.1	F: CGATCTGAACTACATGGTTTAC	128
		R: TCTGCCCATTTGATGTTGC	
*VEGFA*	NM_205042.3	F: CGATGAGGGCCTAGAATGTGTC	101
		R: AGCTCATGTGCGCTATGTGC	
*MMP9*	NM_204667.2	F: ATGAACTACTCCCCCGACCTG	258
		R: AGTCCAGAACTCATCATCATCG	
*IL-1* *β*	NM_204524.1	F: ACTGGGCATCAAGGGCTA	131
		R: GGTAGAAGATGAAGCGGGTC	
*IL-2*	NM_204153.2	F: CGAAGCAAGCAAACAATTCA	151
		R: ATGGTGCCAGTGGTAGGAAG	
*IL-4*	NM_001007079.2	F: TCTTCCTCAACATGCGTCAG	155
		R: TGGTGGAAGAAGGTACGTAGG	
*IL-6*	NM_204628.1	F: CGCCCAGAAATCCCTCCTC	152
		R: AGGCACTGAAACTCCTGGTC	
*IL-10*	NM_001004414.2	F: GCTGCCAAGCCCTGTT	126
		R: CCTCAAACTTCACCCTCA	
*TGF-* *β* *3*	NM_205454.1	F: CATCGAGCTCTTCCAGATCC	111
		R: ACATCGAAGGACAGCCACT	
*TGF-* *β1*	NM_001318456.1	F: AGGATCTGCAGTGGAGTGGAT	168
		R: CCCCGGGTTGTGTTGGT	
*TNF-* *α*	JN942589.1	F: ACCCCTACCCTGTCCCAC	228
		R: GCCAAGTCAACGCTCCTG	
*RUNX2*	NM_204128.2	F: GGCTGGGAACGACGAGAACTAC	190
		R: CGTCACCTTTATGGCTCTGTGG	
*BMP2*	NM_001398170.1	F: CAGCGTAAGCGCCACAAATACA	151
		R: TTAGGTGATCTGCCAGCGGAAA	
*CTSK*	NM_204971.3	F: GATGCCAGTCTGCCCTCCTT	129
		R: CCAGTGCTTGGTGCCCTTCT	
*OPG*	NM_001033641.2	F: AGACTGGAACAGCAACGACGAG	266
		R: GACAGACTGCTTTGGATGACGT	
*RANKL*	NM_001083361.2	F: AGGAGGTGAAGTTAATGCCAGAAT	298
		R: AGTTTCCCATCACTGAACGTCATA	

**Table 3:** Effects of *Dendrobium officinale* leaf supplementation on bone morphology indicators in laying hens ^1^.

Parameter	CON ^2^	DO-L ^2^	DO-H ^2^	SEM	*p* Value
Femur					
Length, mm	86.4	86.0	85.2	0.90	0.65
Width, mm	7.03	7.08	7.29	0.14	0.42
Tibia					
Length, mm	119.5	119.7	119.5	1.40	1.00
Width, mm	6.05	6.22	6.40	0.13	0.16

^1^ Values are least squares means (LSMeans) ± standard error of the mean (SEM); n = 8 hens per treatment. ^2^ CON: control; DO-L: 1200 mg/kg *Dendrobium officinale* leaf; DO-H: 3600 mg/kg *Dendrobium officinale* leaf.

**Table 4:** Effects of *Dendrobium officinale* leaf supplementation on bone density in laying hens ^1^.

Parameter	CON ^2^	DO-L ^2^	DO-H ^2^	SEM	*p* Value
Femur					
Bone mineral content, g	2.88	4.70	4.62	0.60	0.08
Bone density, g/cm^2^	0.32	0.51	0.49	0.16	0.08
Bone area, cm^2^	9.02	9.12	9.31	0.19	0.53
Tibia					
Bone mineral content, g	3.50	4.80	4.94	0.53	0.14
Bone density, g/cm^2^	0.29	0.41	0.40	0.04	0.12
Bone area, cm^2^	12.09	11.80	12.36	0.23	0.25

^1^ Values are least squares means (LSMeans) ± standard error of the mean (SEM); n = 8 hens per treatment. ^2^ CON: control; DO-L: 1200 mg/kg *Dendrobium officinale* leaf; DO-H: 3600 mg/kg *Dendrobium officinale* leaf.

**Table 5:** Effects of *Dendrobium officinale* leaf supplementation on bone strength in laying hens ^1^.

Parameter	CON ^2^	DO-L ^2^	DO-H ^2^	SEM	*p* Value
Femur					
Stiffness, N/mm	515.52	564.75	559.53	44.00	0.69
Breaking strength, N	62.38	109.64	100.35	16.88	0.14
Tibia					
Stiffness, N/mm	1877.74	3019.28	2694.60	373.80	0.11
Breaking strength, N	104.47	248.29	199.85	42.99	0.08

^1^ Values are least squares means (LSMeans) ± standard error of the mean (SEM); n = 8 hens per treatment. ^2^ CON: control; DO-L: 1200 mg/kg *Dendrobium officinale* leaf; DO-H: 3600 mg/kg *Dendrobium officinale* leaf.

**Table 6:** Effects of *Dendrobium officinale* leaf supplementation on the expression of bone metabolism–related genes in laying hens ^1^.

Parameter	CON ^2^	DO-L ^2^	DO-H ^2^	SEM	*p* Value
VEGFA	0.92 ^b^	4.09 ^a^	2.94 ^ab^	0.76	0.03
OPG	0.69	1.52	3.03	0.83	0.15
RUNX2	2.87	7.86	1.89	3.62	0.52
BMP2	2.30	18.84	11.39	5.95	0.19
MMP9	1.32	0.56	1.71	0.79	0.46
CTSK	0.99	1.12	1.64	0.48	0.59
RANKL	0.50	2.01	1.66	0.73	0.37
OPG/RANKL	0.85	2.58	1.94	0.97	0.27

^1^ Values are least squares means (LSMeans) ± standard error of the mean (SEM); n = 8 hens per treatment. ^2^ CON: control; DO-L: 1200 mg/kg *Dendrobium officinale* leaf; DO-H: 3600 mg/kg *Dendrobium officinale* leaf. Different letters indicate a significant difference.

**Table 7:** Effects of *Dendrobium officinale* leaf supplementation on cytokine gene expression in laying hens ^1^.

Parameter	CON ^2^	DO-L ^2^	DO-H ^2^	SEM	*p* Value
IL-1β	2.14	3.03	3.11	1.70	0.90
IL-2α	7.02	9.59	2.68	4.88	0.63
IL-4	2.70	10.36	5.04	2.92	0.23
IL-6	5.87	27.01	11.98	7.91	0.19
IL-10	3.68	20.07	11.98	7.13	0.26
TNF-α	2.48	13.44	15.86	6.26	0.27
TGF-β3	1.57	1.21	3.60	0.82	0.10
TGF-β1	1.30 ^b^	1.23 ^b^	2.66 ^a^	0.39	0.02

^1^ Values are least squares means (LSMeans) ± standard error of the mean (SEM); n = 8 hens per treatment. ^2^ CON: control; DO-L: 1200 mg/kg *Dendrobium officinale* leaf; DO-H: 3600 mg/kg *Dendrobium officinale* leaf. Different letters indicate a significant difference.

## Data Availability

Data are contained within the article.

## References

[1] Abraham M.E., Robison C.I., Kim W.K., Regmi P., Karcher D.M. (2023). n-3 essential fatty acid and vitamin D supplementation improve skeletal health in laying hens. Poult. Sci..

[2] Rufener C., Makagon M.M. (2020). Keel bone fractures in laying hens: A systematic review of prevalence across age, housing systems, and strains. J. Anim. Sci..

[3] Sinclair-Black M., Garcia R.A., Ellestad L.E. (2023). Physiological regulation of calcium and phosphorus utilization in laying hens. Front. Physiol..

[4] Chen W., Lu J., Zhang J., Wu J., Yu L., Qin L., Zhu B. (2021). Traditional Uses, Phytochemistry, Pharmacology, and Quality Control of *Dendrobium officinale* Kimura et. Migo. Front. Pharmacol..

[5] Li J., Tao W., Zhou W., Xing J., Luo M., Yang Y. (2024). The comprehensive analysis of gut microbiome and spleen transcriptome revealed the immunomodulatory mechanism of *Dendrobium officinale* leaf polysaccharide on immunosuppressed mice. Int. J. Biol. Macromol..

[6] Duan H., Yu Q., Ni Y., Li J., Yu L., Yan X., Fan L. (2024). Synergistic anti-aging effect of *Dendrobium officinale* polysaccharide and spermidine: A metabolomics analysis focusing on the regulation of lipid, nucleotide and energy metabolism. Int. J. Biol. Macromol..

[7] Wang Q., Zi C.T., Wang J., Wang Y.N., Huang Y.W., Fu X.Q., Wang X.J., Sheng J. (2017). *Dendrobium officinale* Orchid Extract Prevents Ovariectomy-Induced Osteoporosis in Vivo and Inhibits RANKL-Induced Osteoclast Differentiation in Vitro. Front. Pharmacol..

[8] Wang Y., Jiang Z., Deng L., Zhang G., Xu X., Alonge E., Zhang H., Guo C. (2023). *Dendrobium officinale* polysaccharides prevent glucocorticoids-induced osteoporosis by destabilizing KEAP1-NRF2 interaction. Int. J. Biol. Macromol..

[9] Li X., Gao C., Zhou K., Gan K., Ye T., Zhao J., Li J., Ma C. (2024). Dendrobine ameliorates glucocorticoid-induced osteoporosis by promoting osteogenesis through JNK/p38 MAPK pathway activation and GR nuclear translocation inhibition. J. Agric. Food Chem..

[10] Gang R., Youting C., Jinbao Y., Guoyue Z., Chuanyun X., Wenzan D., Yunlong C. (2020). Phytochemical investigation of leaves of *Dendrobium officinale*. Chin. Herb. Med..

[11] Xie H., Fang J., Farag M.A., Li Z., Sun P., Shao P. (2022). *Dendrobium officinale* leaf polysaccharides regulation of immune response and gut microbiota composition in cyclophosphamide-treated mice. Food Chem. X.

[12] Hu Z.Y., Zhang H.X., Liu Y.L., Ge W., Wang H., Jin H.F., He K., Yan F.F., Zhao A.Y. (2024). Effects of dry powder of *Dendrobium officinale* leaf on production performance, egg quality, serum biochemical indexes, hormone level and organ index of Jinghong No. 1 laying hens. Acta Ecol. Anim. Domastici.

[13] Yao W., Zhang H., Jiang X., Mehmood K., Iqbal M., Li A., Zhang J., Wang Y., Waqas M., Shen Y. (2018). Effect of total flavonoids of rhizoma drynariae on tibial dyschondroplasia by regulating BMP-2 and Runx2 expression in chickens. Front. Pharmacol..

[14] Steinerova M., Horecky C., Nedomova S., Slama P., Vevera P., Tokarcikova K., Pavlik A. (2025). Analysis of selected polymorphisms found within genes involved in bone metabolism signaling pathways with morphometric parameters of bone tissue in broilers. J. Microbiol. Biotechnol. Food Sci..

[15] Liu Y., Liu Q., Yin C., Li Y., Wu J., Chen Q., Yu H., Lu A., Guan D. (2022). Uncovering hidden mechanisms of different prescriptions treatment for osteoporosis via novel bioinformatics model and experiment validation. Front. Cell Dev. Biol..

[16] Webster A.B. (2004). Welfare implications of avian osteoporosis. Poult. Sci..

[17] Zhao S.C., Teng X.Q., Xu D.L., Chi X., Ge M., Xu S.W. (2020). Influences of low level of dietary calcium on bone characters in laying hens. Poult. Sci..

[18] Zhang J., Zhu Y., Zhu Y.L. (2019). Study on the Difference of Immunomodulatory Effects of *Dendrobium officinale* and Its Lyophilized Power on Immunosuppressed Mice. Anhui Agric. Sci..

[19] Yin L., Sun C., Zhang J., Li Y., Wang Y., Bai L., Lei Z. (2025). Critical signaling pathways in osteoclast differentiation and bone resorption: Mechanisms and therapeutic implications for periprosthetic osteolysis. Front. Cell Dev. Biol..

[20] Grosso A., Burger M.G., Lunger A., Briquez P.S., Mai F., Hubbell J.A., Schaefer D.J., Banfi A., Di Maggio N. (2023). VEGF dose controls the coupling of angiogenesis and osteogenesis in engineered bone grafts. npj Regen. Med..

[21] Zhu G., Chen W., Tang C.Y., McVicar A., Edwards D., Wang J., McConnell M., Yang S., Li Y., Chang Z. (2022). Knockout and double knockout of Cathepsin K and Mmp9 reveals a novel function of Cathepsin K as a regulator of osteoclast gene expression and bone homeostasis. Int. J. Biol. Sci..

[22] Han L., Fang W., Jia H. (2020). Effect of *Dendrobium candidum* polysaccharide on RANKL-induced differentiation of mouse bone marrow macrophages into osteoclasts. Acta Univ. Med. Anhui.

[23] Xu J., Yu L., Liu F., Wan L., Deng Z. (2023). The effect of cytokines on osteoblasts and osteoclasts in bone remodeling in osteoporosis: A review. Front. Immunol..

[24] Chiba-Ohkuma R., Karakida T., Yamamoto R., Yamakoshi Y. (2025). Direct and indirect effects of transforming growth factor-beta on osteoclast-mediated bone remodeling using a new in vitro bone matrix model. JBMR Plus.

[25] Zhu C., Ding X., Chen M., Feng J., Zou J., Zhang L. (2025). Exercise-mediated skeletal muscle-derived IL-6 regulates bone metabolism: A new perspective on muscle-bone crosstalk. Biomolecules.

[26] Zhang L., Peng H., Xu J., Xu Y., Yin Y., He B., Zhuang J. (2019). Effects of *Dendrobium officinale* polysaccharides on brain inflammation of epileptic rats. Oxid. Med. Cell Longev..

